# Genetic and genomic resources for *Rubus* breeding: a roadmap for the future

**DOI:** 10.1038/s41438-019-0199-2

**Published:** 2019-10-15

**Authors:** Toshi M. Foster, Nahla V. Bassil, Michael Dossett, Margaret Leigh Worthington, Julie Graham

**Affiliations:** 1 The New Zealand Institute for Plant and Food Research (PFR) Ltd, 55 Old Mill Road, Motueka, New Zealand; 2 USDA ARS National Clonal Germplasm Repository (NCGR), 33447 Peoria Rd., Corvallis, OR USA; 3 Blueberry Council (in Partnership with Agriculture and Agri-Food Canada) Agassiz Food Research Centre, Columbia, BC V0M 1A0 Canada; 4 Department of Horticulture, University of Arkansas, 316 Plant Science Building, Fayetteville, AR 72701 USA; 5 The James Hutton Institute, Errol Road, Invergowrie, Dundee, DD2 5DA Scotland

**Keywords:** Plant breeding, Plant physiology

## Abstract

*Rubus* fruits are high-value crops that are sought after by consumers for their flavor, visual appeal, and health benefits. To meet this demand, production of red and black raspberries (*R. idaeus* L. and *R. occidentalis* L.), blackberries (*R*. subgenus *Rubus*), and hybrids, such as Boysenberry and marionberry, is growing worldwide. *Rubus* breeding programmes are continually striving to improve flavor, texture, machine harvestability, and yield, provide pest and disease resistance, improve storage and processing properties, and optimize fruits and plants for different production and harvest systems. Breeders face numerous challenges, such as polyploidy, the lack of genetic diversity in many of the elite cultivars, and until recently, the relative shortage of genetic and genomic resources available for *Rubus*. This review will highlight the development of continually improving genetic maps, the identification of Quantitative Trait Loci (QTL)s controlling key traits, draft genomes for red and black raspberry, and efforts to improve gene models. The development of genetic maps and markers, the molecular characterization of wild species and germplasm, and high-throughput genotyping platforms will expedite breeding of improved cultivars. Fully sequenced genomes and accurate gene models facilitate identification of genes underlying traits of interest and enable gene editing technologies such as CRISPR/Cas9.

## Introduction

*Rubus* is a large and diverse genus in the *Rosoideae* subfamily of Rosaceae, with over 740 species described worldwide^1^. Based on phenotypic diversity, it is thought that *Rubus* originated in southwestern China^2^. Pliny the Elder (AD 45) wrote about the people of Troy gathering “ida fruits” (red raspberries) at the base of Mount Ida in what is now Turkey. Although there are species native to most temperate regions, they are also found from the sub-tropics to Arctic regions and can grow from sea level to 4500 m^3^.

The genus is divided into 12 subgenera^4^. There is a wide spectrum of wild species, but the focus for domestication and breeding, and the most economically important crops are red and black raspberry (*R. idaeus* L. and *R. occidentalis* L., both in subgenus *Idaeobatus*), and blackberry (*R*. subgenus *Rubus*). Raspberries are diploid (2*n* = 2 × = 14), and blackberries range from diploid to 12 × (2*n* = 2 × = 14 to 2*n* = 12 × = 84). Red and black raspberries readily hybridize to produce purple raspberries. Generally, blackberry cultivars are not assigned a species, as there are several species in the ancestry of all the cultivars. Similarly, *R. idaeus* and several other species hybridize with the blackberry species to produce fertile accessions. Natural and human made hybrids are common within *Rubus*. This review will focus primarily on the past, present, and future of genetic and genomic tools to facilitate the improvement of raspberries and blackberries.

Prior to domestication, the primary use of *Rubus*, especially blackberry, was medicinal and they were foraged by indigenous communities. There are records of the root, leaves, stem, and fruits being used to treat a variety of ailments^5^. More recently, *Rubus* fruits were found to be very high in secondary metabolites, such as anthocyanins and phenolics, that provide antioxidant capacity, supporting their reputation as “superfoods”^6–10^. Blackberries are particularly high in dietary fiber, vitamins C and K, and manganese^11^.

Modern cultivars are bred for the fresh market and processing (e.g., freezing, drying, canning), and for the home garden. In 2017, world production of red and black raspberries reached 840,000 tonnes, with Europe and the Americas being the top producers^12^. A survey of global blackberry production in 2005 indicated that the two largest production regions were North America (59,123 tonnes) and Europe (43,000 tonnes)^13^. During the past 14 years, since the last worldwide survey was conducted, the global blackberry industry has experienced rapid change and growth. This growth has been driven by increased consumer demand, new cultivars, advanced production methods, and year-round product availability. Blackberries are currently the fourth most economically important U.S. berry crop, accounting for $549 million in sales during 2016^14^. The Mexican fresh-market blackberry industry, in particular, has rapidly expanded, with almost 11,000 ha planted in 2015 and most fruit destined for export to the USA and other markets^15,16^. Fresh-market blackberry production is also growing in other regions including Southern Europe, Australia, and Central and South America.

There are a number of *Rubus* breeding programs worldwide. General breeding targets include high fruit quality and yield, extended cropping season, good storage and processing properties, disease and pest resistance, and adaptation to local growing environments. Other desired traits are specific for the fresh market, processing, and ornamental cultivars.

### *Rubus* life cycle and physiology

The plants, commonly known as brambles or caneberries, generally grow as a deciduous shrub with biennial canes that are initiated from a perennial root system. The canes often have epidermal spines, ranging from hair-like to sharp thorns. Growth forms can be erect, trailing, or more vine-like. *Rubus* can propagate sexually, by apomixis, and vegetatively, enabling them to be highly invasive.

Most *Rubus* plants are biennial-fruiting (BF) (also called floricane-fruiting or summer-fruiting); these initiate axillary floral buds toward autumn of the first year of growth, but the buds do not develop into fruits until spring/summer of the following year. Annual-fruiting (AF) cultivars of both raspberry and blackberry (also called primocane-fruiting or autumn-fruiting) initiate flowers in late spring/early summer and these develop into fruits from summer until late autumn of the same year. In both types, flowering and fruiting initiates from the shoot tip and develops basipetally after vegetative growth has stopped. The key developmental difference between the two flowering phenologies is that AF floral buds are initiated earlier and progress directly to fruit set, whereas floral initiation is followed by dormancy in BF types^17–22^.

Based on a number of studies, floral induction in BF cultivars is triggered by a combination of decreased temperatures and shorter photoperiod^18,19,21,23^. While there is no absolute requirement for AF cultivars to experience chilling in the season prior to initiate flowering, as newly initiated canes can progress through to fruiting in a single season, the expression of AF in terms of floral consistency across canes and the total number of flowers is strongly influenced by chilling^17,24^. There is also evidence of a short juvenile phase of 15 or more vegetative nodes before plants are able to flower, even under inductive conditions^19,21^.

**Fig. 1:**
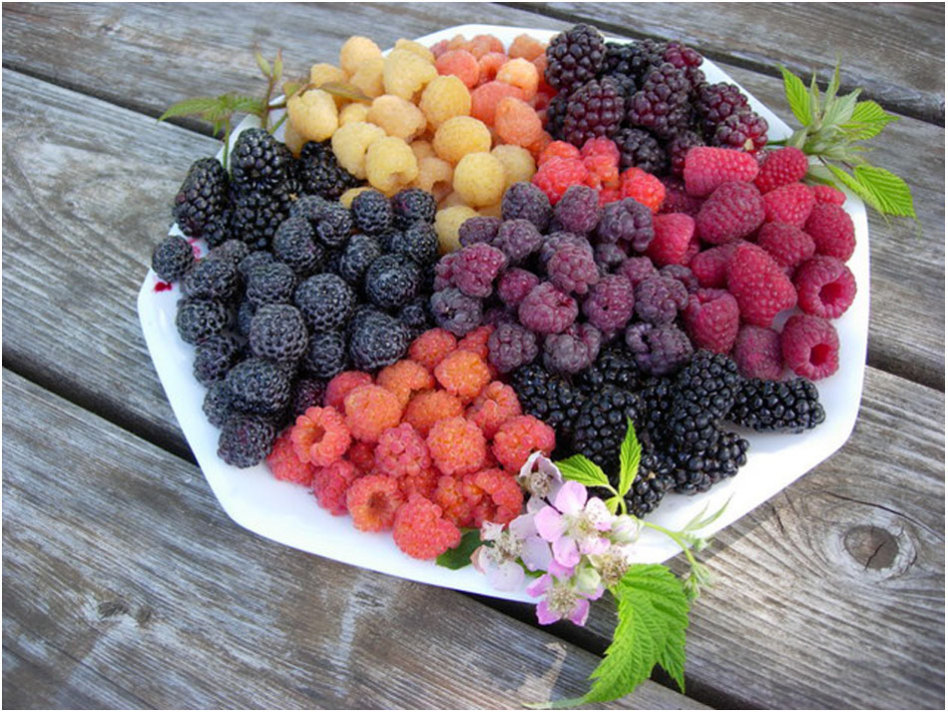
Photograph illustrating the wide diversity in color, size, and shape of *Rubus* fruits

*Rubus* fruits are an aggregate of small fleshy drupelets, each containing a single seed derived from a fertilized ovary. The fruit takes 35–45 days to develop and can be germinated after scarification and a short period of stratification. At maturity raspberries detach from the receptacle, whereas blackberries and many hybrids do not, and the receptacle is picked with the fruit. Figure 1 shows a range of *Rubus* fruits.

### Genetic markers for faster breeding

Relative to other crops, there have been few genetic and genomic resources available for *Rubus* improvement until recently. For raspberry, the lack of genetic diversity found in most of the elite cultivars presents another challenge^25^. The development of high-density genetic maps and markers, the molecular characterization of more wild species and germplasm, and high-throughput genotyping platforms will expedite breeding of improved cultivars.

Early DNA markers, such as minisatellites, restriction fragment length polymorphisms (RFLPs), random amplified polymorphic DNA (RAPDs), amplified fragment length polymorphisms (AFLPs), internal transcribed spacer region (ITS), and their use in *Rubus* have been reviewed by others^26,27^. Newer markers such as simple sequence repeat (SSR), also known as microsatellites, and single nucleotide polymorphism (SNP) markers are available for *Rubus*. Microsatellite-enriched libraries were first used to develop a limited number of genomic SSR markers in *R. alceifolius*^28^, *R. idaeus* ‘Glen Moy’^29–31^ and ‘Meeker’^32^, *R. hochstetterorum*^33^, blackberry ‘Marion’^32^, *R. glaucus*^34^, and *R. coreanus*^35^.

Expressed sequence tags (ESTs) later provided a good source of EST-SSR markers and were developed from: red raspberry “Glen Moy”^29^, “Glen Ample”, “Latham”^36^, and “Heritage”;^37^ the black raspberry “Bristol”;^38^ and the blackberry “Merton Thornless”^39^. Next-generation sequencing was also used to develop SSR sequences from short reads of red and black raspberry^40^. Additional SSRs and SNP markers were also developed from transcriptome sequences of ‘Boysen’^41^ and “Loch Ness”^42^ blackberries and from candidate gene sequences^43–47^, most of which were identified from Bacterial Artificial Chromosome (BAC) sequences of “Glen Moy” red raspberry and used for linkage mapping. Microsatellite markers have been used for fingerprinting, cultivar identification, or pedigree confirmation^25,32,48–52^, assessment of genetic diversity^25,53–60^, in addition to linkage mapping and QTL analyses^29,30,36,38,43–47,61–70^.

### QTLs/loci controlling traits of interest

Graham et al.^29^ created the first genetic linkage map from a “Glen Moy” × “Latham” red raspberry population, and this map has subsequently been improved using genotyping by sequencing (GBS)^71^. Analysis of this population has identified numerous QTLs affecting fruit ripening traits, such as fruit texture, color, anthocyanin accumulation, flavor volatiles and fruit softening^29,30,43,44,47,63–65,72^. Genes in the phenylpropanoid pathway and candidate genes in the biosynthesis of flavor volatiles have been mapped to these loci^45–47^. Two QTLs that are important for the genetic control of the crumbly fruit disorder were identified^65^. For a recent and comprehensive review of QTL and trait mapping, see McCallum et al.^73^.

The genetic regulation of annual vs biennial fruiting has been the subject of multiple studies^62,74–81^. Castro et al.^62^ reported mapping a single locus on LG7 conferring AF in blackberry. However, in this study LG7 was assigned by default and none of the LG7 markers mapped to the black raspberry genetic map of Bushakra et al.^61^. More recently, two additional loci conferring AF have been identified on LG3 and LG4 of red raspberry using a high-density map generated by GBS-based SNP markers (Jibran et al.^89^, paper submitted).

Several QTLs for cane splitting have been identified^67^, with two of these co-locating with a previously identified QTL for plant vigor and another associated with a QTL for resistance to root rot^64^. Gene H, which controls cane pubescence and is associated with resistance to cane botrytis and spur blight has been mapped to LG2^30^. Three other loci were identified that are associated with resistance to rust and cane spot, and spine density^30^. Resistance to raspberry aphids has been mapped to two loci on LG3 and LG6^38,66^. QTLs for root vigor and resistance to phytophthora root rot have been identified^64^ and markers from these deployed by the James Hutton Institute Raspberry Breeding Consortium. QTLs relating to physical traits that affect pest burden also have been identified^63^. Recently, attempts have been made to map QTLs from hyperspectral traits in an attempt to develop high-throughput phenotyping approaches^82^.

At least six different types of dwarf raspberry^83,84^ and one brachytic dwarf blackberry^85^ have been described. One dwarf raspberry locus has been mapped to LG6^66^ and another to the bottom of LG2 (Jibran et al.^89^, unpublished data), suggesting that multiple loci can confer a dwarf habit.

The first tetraploid blackberry genetic linkage map was constructed from a full-sib family of “Prime-Jim” × “Arapaho” that was segregating for thornlessness and AF^62^. One hundred and nineteen SSR markers were used to create an integrated linkage map composed of seven linkage groups and to map loci for thornlessness and AF to LG4 and 7, respectively. A second tetraploid blackberry linkage map was constructed also using simplex markers consisting of restriction-site associated genomic DNA (RAD-Seq) in the tetraploid “Chester Thornless” × “Prime-Jim” population^86^. Parental haplotype maps for “Chester Thornless” and “Prime-Jim”, consisted of 29 linkage groups spanning 1059 cM and 31 linkage groups spanning 1025 cM, respectively; which provided supporting evidence for the position of the thornless locus on LG4. No broadly predictive markers were identified for thornlessness or AF in either of these mapping studies and molecular markers have yet to be used for any application other than parentage confirmation in applied blackberry breeding programs.

### Genomes sequenced

Next-generation sequencing techniques in addition to long-read PacBio sequencing and Hi-C scaffolding have been used to generate a chromosome-scale genome assembly of a highly homozygous wild accession (ORUS 4115–3) of black raspberry^87–89^. The V3 reference genome has a contig N50 of 5.1 Mb, consists of 235 contigs that were anchored and oriented into seven chromosomes, and contains 47 Mb of new sequences including large pericentromeric regions and thousands of previously unannotated protein-coding genes.

A genome assembly of “Heritage” red raspberry has been reported^90,91^ but is not publicly available, while a fragmented short-read-derived draft assembly of “Glen Moy” and “Latham” was recently generated^71^. This draft assembly of 147,546 scaffolds covers 361,105,105 bp of the estimated 280 Mb genome. Comparison against plant near-universal single-copy orthologs using Benchmarking Universal Single-Copy Orthologs (BUSCO) indicated that over 90% of the 1440 BUSCO groups were present in the genome assembly. A new genome assembly of “Joan J” red raspberry has recently been generated using a combination of long single-molecular real-time (SMRT) PacBio reads and high coverage short reads^92^. This draft genome is 299 Mb, consists of 2145 scaffolds and has a BUSCO genome completeness of 95.3% and an N50 score of 638 Kb.

A team of researchers is developing reference genomes for two diploid blackberry accessions representing the sources of thornlessness (“Burbank Thornless”, *R. ulmifolius inermis*) and AF (“Hillquist”, *R. argutus*) in fresh-market blackberry breeding programs^93^.

### High-throughput genotyping

At this time, the only high-throughput genotyping methods used in *Rubus* consist of reduced representation sequencing techniques such as target capture sequencing for phylogenetic analyses^94^, GBS for developing saturated linkage maps in black raspberry^38^ and in red raspberry^71,90^, and RAD-Seq for linkage mapping in blackberry^95^. Target capture was applied to 96 samples that included representatives from each of the 12 *Rubus* subgenera and from five known hybrids or economically important cultivars. The target capture baits included 926 single-copy loci from the *R. occidentalis* genome and 247 loci that are conserved among apple, peach and strawberry genomes^94^. Preliminary analyses indicated that the subgenus containing raspberries, *Idaeobatus*, was polyphylet with members occurring in five out of eight clades. The phylogenetic network identified a number of polyploid taxa as potential hybrids, indicated by their intermediate position between two major clades. “Marion”, “Logan”, and “Boysen”, which have multiple raspberry and blackberry species in their pedigrees, were located between the raspberry and blackberry clades^94^. The Elshire et al.^96^ method of digestion with ApeK1 was used by Bushakra et al.^38^ and Ward et al.^90^, while the two-enzyme method of Poland et al.^97^ was used by Hackett et al.^71^, and these resulted in highly saturated GBS-based linkage maps.

### Anchoring physical and genomic maps, and synteny across *Rosaceae*

Linkage maps have been constructed for red raspberry^29,30,36,43–47,63–66,68–71,90^, black raspberry^38^, a cross between red and black raspberry^61^, and blackberry^62,86,98^. Bushakra et al.^61^ aligned the genetic map of the red raspberry parent, “Latham”, to BLAST-generated physical maps of *F. vesca* “Hawaii 4”^99^, *Malus* × *domestica* “Golden Delicious”^100^ and *Prunus persica*, “Lovell”^101^, and to the nine hypothetical *Rosaceae* ancestral chromosomes^102^, using sequence-based orthologous markers in common among them. The 1:1 collinearity of the seven “Latham” linkage groups to the seven *Fragaria* chromosomes led the authors to rename the groups to correspond to those used in *Fragaria* as *Rubus* Linkage Groups (RLGs) 1 through 7. This nomenclature was subsequently used in red raspberry^90^, in black raspberry^38^ and in blackberry^62^. Each of the seven linkage groups in *Rubus* were aligned to 1, 2 or 3 segments of the *Malus* and the *Prunus* genomes. Of these four *Rosaceae* genera, *Prunus* appears to show the fewest rearrangements from the proposed ancestral state.

**Fig. 2:**
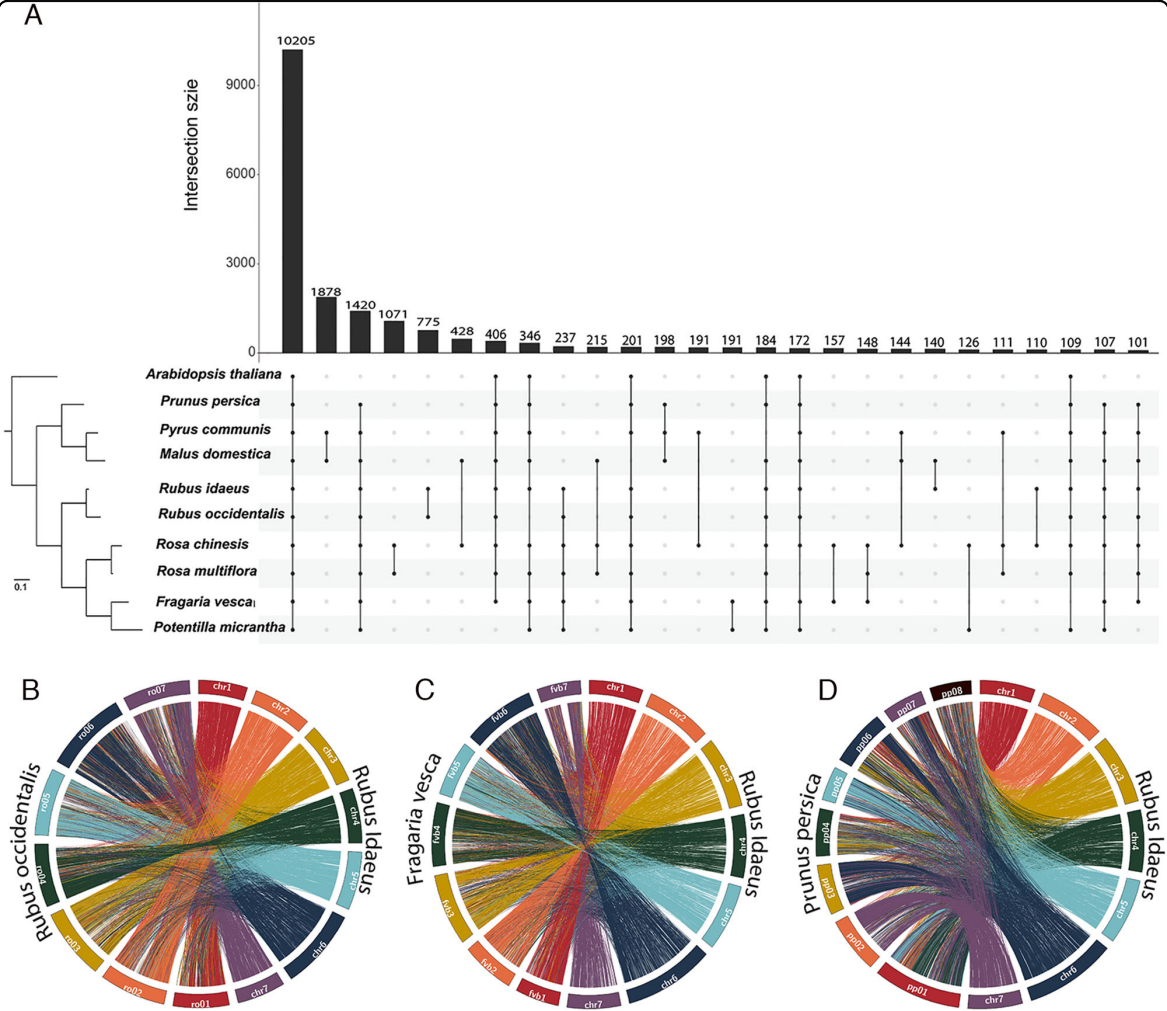
Circos plots displaying macrosynteny between the genomes of **a** *Rubus idaeus* and *Rubus occidentalis*, **b** *R. idaeus* and *Fragaria vesca*, and **c** *R. idaeus* and *Prunus persica*. For A to D, each connecting line represents an orthologous gene pair and the right half of each circle consists of the seven *R. idaeus* chromosomes colored by the spectral order in the rainbow. Reproduced with permission from Wight et al.^92^

The pseudomolecules making up the V3 black raspberry genome assembly (Ro01-Ro07) were anchored to the seven haploid strawberry chromosomes (Fvb1–7) using markers from GBS-based genetic maps^88^ and confirmed a high degree of synteny across both genomes despite the 75 MY divergence. No major rearrangements were observed when comparing Ro01/Fvb1, Ro02/Fvb2, and Ro03/Fvb3, while the other four chromosome pairs had one or two major inversions. The black raspberry and *F. vesca* genomes had 15,727 syntenic gene pairs, and each genome had unique patterns of expansion/deletion based on gene-level microsynteny. VanBuren et al.^88^ suggested that differences in gene composition between these two species are due to a combination of tandem gene duplications, retrotransposon-mediated duplication/movement, fractionation/deletion, and mis-annotation. Most recently, genome-wide comparisons between *R. idaeus*, *R. occidentalis*, and nine other *Rosaceae* species have supported the high collinearity between raspberry and strawberry genomes^92^. Peach (*Prunus persica*) has slightly less collinearity with raspberry, although there are large conserved syntenic blocks (Fig. 2).

### Transcriptomic analysis

Eight RNAseq datasets (leaves, stems, canes, green fruit, red fruit, ripe fruit and root tissue harvested from *Verticillium dahlia*-treated and control plants two months after inoculation) of “Jewel” generated 28,005 protein-coding genes in the V1 black raspberry assembly^87^, while another eight RNAseq libraries (leaves, methyl jasmonate-treated leaves, flowers, canes, roots, green fruit, red fruit, and ripe fruit) from ORUS 4115–3 resulted in 34,545 high-confidence gene models in the V3 genome assembly^88^. The V3 annotation had 9301 new gene models that were improved or absent in the V1 assembly, while 4020 low-quality gene models from V1 were removed from V3 because of insufficient protein support of transposable element-related annotation. Surveying expression in ripening fruit of “Jewel” identified 4446 genes that were differentially expressed between green and ripe fruit, and 8376 genes between red and ripe fruit^87^. The genes that were upregulated during fruit ripening consisted of those involved in hydrolase activity, cell wall degradation, sugar transport, and anthocyanin accumulation and biosynthesis, among others. Genes involved in anthocyanin accumulation during fruit ripening were also previously identified in what was erroneously labelled *R. coreanus*^103^ and is, in fact, *R. occidentalis*^104^. A type I chalcone Isomerase, RcMCHI2 (Unigene 18325), from *R. coreanus* complemented an *Arabidopsis* testa 5–1 mutant and restored its ability to produce anthocyanin pigments in the cotyledon and hypocotyl and to accumulate delphinidin 3-O-rutinoside and cyaniding 3-O-rutinoside. Recently, differential expression was evaluated in fruit transcripts of *R. coreanus* from China across three developmental stages^105^. They identified 23 transcripts in the flavonoid biosynthesis pathways whose expression corresponded to metabolite accumulation during ripening. Seven representative genes were validated by sequencing after cloning, and their expression was confirmed by RT-qPCR. In that study, they also identified 119 nucleotide-binding site leucine-rich repeat (NBS-LRR) protein-coding genes. A red raspberry “fruit transcriptome” comprising a database of 56,000 unigenes has been established and mapped to the genome scaffolds of “Glen Moy” (Milne, personal communication).

More recently, a red raspberry transcriptome was generated from 18 RNAseq libraries derived from fruit tissues at early stages of development^92^. A total of 35,566 protein-coding genes were annotated. Interestingly, 775 orthogroups were limited to the *Rubus* genera, and these were significantly enriched with genes involved in defense and gene regulation.

In the V1 gene models of black raspberry, 144 predicted genes contained motifs that were conserved among disease resistance R genes and 10 putative homologues to the tomato Ve1 gene that confers *Verticillium* wilt (VW) resistance^87^. Up to 147 genes, including eight with homology to genes associated with disease resistance and 12 with homology to transcription vectors, were differentially expressed between *V. dahlia*-inoculated and control “Jewel” roots^38,87^. More work is needed to identify the genes responsible for VW resistance.

### Resources for blackberry

Genetic and genomic resources in blackberry have been delayed by challenges including polyploidy, multisomic inheritance, and heterozygosity. The development of a chromosome-level genome assembly in black raspberry and a new sequencing initiative with two diploid relatives in the subgenus *Rubus* will facilitate rapid advances in blackberry. Software has recently been developed for linkage and QTL mapping^106,107^, association analyses^108^ and genomic selection^109^ in multisomic polyploid species. These new software packages all require large datasets of SNP markers with accurate dosage calls, e.g. AAAT, AATT, ATTT, in heterozygous individuals. Software is available for estimating allelic dosage from a fixed genotyping platform, e.g., SNP chip^110,111^; however, there is currently no *Rubus* chip. Dosage estimation is significantly more difficult in GBS datasets because of problems of missing data and uneven depth of coverage. Fortunately, GBS strategies that severely restrict genome complexity^112^ and sequence capture methods^113^ have allowed the generation of large quantities of markers with sufficient read depth to estimate allele dosage in polyploid crop species lacking fixed SNP arrays. In the near future, these new *Rubus* genomic resources coupled with development of new software for analyzing genomes of polyploid plants will enable blackberry researchers to execute QTL mapping and genome-wide association studies (GWAS) for important breeding traits.

### Future perspectives

Driven by consumer demand for high-health and delicious flavor, berryfruit sales have increased steadily in the past decade and are projected to grow in the future. There is also an increased demand from consumers for sustainably produced, pesticide-free and locally grown fruits. While in conflict with consumers’ desire for decreased plastic use, growers worldwide are increasingly moving towards production in containers under poly-tunnel houses to lengthen the production season, to reduce water and chemical inputs, and to protect from adverse weather. These new production systems bring new challenges that will change breeding targets. Climate change creates another set of problems for some cultivars, with many areas no longer receiving sufficient or predictable winter chill. Recent advances in genomic tools for *Rubus* will help accelerate breeding new cultivars optimized for the changing environment^93,114^.

New resources could be developed that would further fast-track both breeding and basic science discovery. The *Rubus* germplasm is incredibly variable and offers an excellent source of new traits and resistance to pests and diseases. Molecular and phenotypic characterization of existing and novel germplasm would have enormous potential. Tools such as genomic selection have greatly facilitated introducing desirable traits from wild species of apple^115,116^. By coordinating all sequencing data within the *Rubus* community, we could work towards developing a SNP chip for high-throughput genotyping.

Gene models created by computational prediction are often incorrect. Recently, the kiwifruit (*Actinidia chinensis* var. *deliciosa*) Red5 gene models were manually annotated by a consortium of kiwifruit researchers^117^. It was found that 91% of the previous, computationally predicted gene models were incorrect. Moreover, the manual annotation also revealed many translocation events that followed whole-genome duplication and enabled 164 Mb of previously unassigned sequence to be placed on chromosomes. A community annotation approach for raspberry would be relatively simple given the much smaller size of the raspberry genome. Improving the gene models will help with gene identification, allele mining, and with understanding the molecular basis of specific traits.

*Rubus* has many features that make it an excellent model system for *Rosaceae*. It has a very short juvenile phase, is easy to cross, the fruit develop quickly and produce many seeds. Being diploid, raspberry is well suited for genetic studies. Raspberry and blackberry are amenable to *Agrobacterium*-mediated transformation, enabling testing of gene function and gene editing using CRISPR/Cas9 systems^118,119^.

Although *Rubus* has somewhat lagged behind other crops in terms of having genetic and genomic tools, we can catch up rapidly by adopting the most successful strategies and by working together as a community.
